# The RNA m^5^C methyltransferase NSUN1 modulates human malaria gene expression during intraerythrocytic development

**DOI:** 10.3389/fcimb.2024.1474229

**Published:** 2024-10-07

**Authors:** Ruoyu Tang, Yanting Fan, BinBin Lu, Qunfeng Jiang, Xinyu Cheng, Zuping Zhang, Li Shen, Xiaomin Shang

**Affiliations:** ^1^ Department of Parasitology, Xiangya School of Medicine, Central South University, Changsha, China; ^2^ Key Laboratory of Spine and Spinal Cord Injury Repair and Regeneration of Ministry of Education, Tongji Hospital, Clinical Center for Brain and Spinal Cord Research, School of Medicine, Tongji University, Shanghai, China; ^3^ Department of Parasitology, School of Medicine, Northwest University, Xi’an, Shanxi, China; ^4^ Laboratory of Molecular Parasitology, The Key Laboratory of Arrhythmias of the Ministry of Education of China, Research Center for Translational Medicine, Shanghai East Hospital, Clinical Center for Brain and Spinal Cord Research, School of Medicine, Tongji University, Shanghai, China; ^5^ Department of General Manager Office, Hunan Xingchen Biotechnology Company, Yongzhou, China

**Keywords:** *Plasmodium falciparum*, 5-methylcytosine, RNA modification, regulation, 28S ribosomal RNA

## Abstract

**Introduction:**

*Plasmodium falciparum* is the most damaging malaria pathogen and brings a heavy burden to global health. Host switching and morphological changes in *P. falciparum* are dependent on an effective gene expression regulatory system. C5 methylation of cytosines is a common RNA modification in eukaryotes, and the NSUN family are essential m^5^C modification executors. Currently, little is known about this family in *Plasmodium* spp. In this study, we focus on exploring the function of *PfNSUN1* protein.

**Methods:**

An efficient CRISPR/Cas9 gene editing technique was applied to construct the *PfNSUN1* knockdown strain. The knockdown efficiency was confirmed by growth curves and western blot experiments. The knockdown transcriptome data was acquired to find differentially expressed genes, and target genes of *PfNSUN1* protein were identified by RNA immunoprecipitation and high-throughput sequencing experiments.

**Results:**

The efficiency of *PfNSUN1* protein down-regulated was about 34%. RNA-seq data revealed that differentially expressed genes were mainly down-regulated. And there were 224, 278, 556 genes that were down-regulated with more than 2-fold changes and p-adj<0.05 at ring, trophozoite and schizont stages, respectively. *PfNSUN1* protein was significantly enriched on 154 target genes, including 28S ribosomal RNA and *pfap2-g5* transcription factor.

**Discussion:**

*PfNSUN1* is a crucial RNA post-transcriptional modification protein in *P. falciparum*. It plays a pivotal role in regulating gene expression and parasite growth by targeting 28S ribosomal RNA and *pfap2-g5* transcription factor.

## Introduction

Malaria is an important life-threatening parasitic disease, with 249 million people still infected and about 608,000 deaths worldwide in 2022. Cerebral malaria caused by *Plasmodium falciparum* is the leading contributor to malaria deaths (Muppidi et al., 2023). The parasite has a complex life cycle, which amplifies one generation every 48 h in human erythrocytes. Such rapid morphological transformation requires a highly regulated gene expression mechanism.

Posttranscriptional modification of RNA has emerged as a crucial mode of gene expression regulation (Zhao et al., 2017; Baumgarten et al., 2019; Barbieri and Kouzarides, 2020; Govindaraju et al., 2020; Sinha et al., 2021; Hao et al., 2022; Wang et al., 2022). Recent studies have revealed that dynamic and reversible RNA modifications are involved in essential biological processes such as cell development, fate determination, pathogen infection, and stress response in eukaryotes (Engel et al., 2018; Frye et al., 2018; Delaunay and Frye, 2019; Wnuk et al., 2020). C5 methylation of cytosines (5-methylcytosine or m^5^C) is a common modification found on various RNA molecules. In eukaryotes, the m^5^C modification is primarily catalyzed by members of the NOL1/NOP2/SUN (NSUN) structural domain methyltransferase family (Bohnsack et al., 2019; Tao et al., 2023). Most NSUN proteins are conserved in functions, and mutations in *nsun* genes have been shown to be associated with a variety of human diseases (Abbasi-Moheb et al., 2012; Khan et al., 2012).

In *P. falciparum*, four members of the NSUN family (NSUN1-4) have been identified, which contain conserved methylation functional domains similar to those observed in other eukaryotes (Liu et al., 2022). Functional studies on *nsun1*, *nsun3*, and *nsun4* have not been reported yet. Nevertheless, disruption of NSUN2 has been demonstrated to impede the development of gametocytes in both *P. falciparum* and *P. yoelii*, and it is a crucial epigenetic regulator during the transmission of malaria (Liu et al., 2022). Therefore, the NSUN family is expected to be a promising target for malaria prevention and deserves to be intensively investigated.

In this study, a series of experiments was conducted to investigate the function of the *PfNSUN1* protein. The results demonstrate that *PfNSUN1* is indispensable for parasite growth and development throughout the intraerythrocytic developmental cycle (IDC). The knockdown of *PfNSUN1* resulted in a number of alterations in gene expression, including several key regulators. In other eukaryotes, the NSUN1 protein has been demonstrated to be a major methyltransferase of ribosomal RNA (rRNA) (Sharma et al., 2013; Liao et al., 2022). It was found that the PfNSUN1 protein was highly enriched on some 28S rRNA genes. This suggests that the role of the *PfNSUN1* protein is well-conserved and linked to ribosome biogenesis. In conclusion, our study demonstrates the crucial function of the *PfNSUN1* protein in parasite growth and offers novel insights for advancing malaria research.

## Materials and methods

### Transgenic line construction

The plasmids used in this experiment were laboratory-modified plasmids as described previously (Shang et al., 2022). The Cas9 gene was carried by *pUF1* plasmid, and the guide RNA (5′-GGTTGTTGGGAAGAAGCAAA-3′) and homologous arm sequences were carried by *pL6cs* plasmid. Plasmid construction is described below. First, the guide RNA was cloned into the *pL6cs* construct between *XhoI* and *AvrII* restriction enzyme sites. Subsequently, the C-terminal homologous arm sequences of *pfnsun1* with the *ty1-glms* tag were cloned into *AflII* and *AscI* restriction enzyme sites. The successfully constructed plasmids were transformed into *Escherichia coli* XL10 for amplification and purification. Primers used to amplify homologous sequences are listed below: *pfnsun1*-5′HR-F: GGA TAA TGC AAT GGA TAC AC; *pfnsun1*-5′HR-R:GTT CAA AAT GTA TGG CAA CAT C; *pfnsun1*-3′HR-F:ACA AAT GGT TCT GGA GGT GAA GCA AAA GGA AAA ATA ATA ATA GAT G; and *pfnsun1*-3′HR-R: ATC CTT TTT ACC AAG CAC TC.

100 μg purified *pL6cs-nsun1-ty1-glmS* plasmids and 100 μg *pUF1-Cas9-BSD* plasmids were electro-transfected into fresh RBCs under the condition of 310V and 950μF, then enriched schizont-stage parasites were added into RBCs. As soon as the parasitemia reached 5% after invasion, selection drugs (2.5 nM WR99210 and 2 μg/ml Blasticidin S deaminase (BSD)) were started to add until live parasites could be found in the Giemsa solution-stained thin blood smears. Then parasites were collected and genomic DNA was extracted for PCR identification and Sanger sequencing. Once the strain was successfully constructed, it was cloned out by limiting dilution cloning as described (Fan et al., 2020).

### Parasite culture

*Plasmodium falciparum* lines 3D7 and *Pfnsun1-ty1-glmS* were cultivated *in vitro* according to standard procedures. In brief, parasites were cultured in complete RPMI 1640 medium (Thermo Fisher Scientific, Carlsbad, CA, USA) containing 0.5% Albumax I (Thermo Fisher Scientific) and grown in media with type O+ erythrocytes at a 2% hematocrit. The incubator was set to a gas phase condition of 5% O_2_, 5% CO_2_, and 90% N_2_ at 37°C. To obtain tightly synchronized parasites, parasites were purified with 70%/40% Percoll–sorbitol gradients at the schizont stage. After schizonts invaded fresh RBCs for about 5 h, the resulting rings were synchronized with 5% sorbitol treatment and used for subsequent research.

### Growth curve analysis

*Pfnsun1-ty1-glmS* strain was tightly synchronized to a 5-hour window as previously mentioned. In brief, parasites were collected by the Pecoll-sorbitol solution at schizont stage, subsequently invading new erythrocytes. 5-6 hours later, ring-stage synchronization was performed using the sorbitol solution. The strictly synchronized parasites were diluted to 0.1% parasitemia at ring stage and cultured in 6-well plate which were divided into 2 groups, i.e. with or without 5 mM glucosamine (GlcN). Parasites were cultured for four consecutive replicating cycles, and Giemsa-stained thin blood smears were collected from each cycle to count parasitemia. After three independent replications of the experiment, the data obtained were graphed using GraphPad Prism9 software.

### Western blotting

The *Pfnsun1-ty1-glmS* strain was tightly synchronized, and ring-stage parasites were diluted to 1% parasitemia with 2% hematocrit in the presence or absence of 5 mM GlcN. Ring, trophozoite, and schizont stage samples were collected for Western blot analysis at the next life cycle. Briefly, infected RBCs were lysed by 0.15% saponin to release parasites, which were washed by PBS and resuspended in an equal volume of 2 × protein loading buffer, then heated at 100°C for 5 min. The samples were stored at – 80°C for later experiments. Proteins were separated by 8%–10% SDS-PAGE according to the molecular weight and transferred to the PVDF membrane. The primary antibodies were incubated for 1 h at RT. After three washes with PBST, membranes were incubated with the secondary antibody for 1 h at RT. After five washes, target protein signals were detected using ECL Western Blotting Kit (GE Healthcare, USA). The primary antibodies used in this study were mouse anti-ty1 (Sigma, Germany) at 1:1,000 and rabbit anti-aldolase (Abcam, England) at 1:2,000. The HRP-conjugated secondary antibodies were goat antimouse IgG (Abcam, England) and goat antirabbit IgG, which were diluted to 1:5,000.

### RNA extraction, library construction, and RNA-seq data analysis

*Pfnsun1-ty1-glmS* parasites were tightly synchronized by Percoll and sorbitol to a 5-h window with or without 5 mM GlcN treatment as described above. Samples were collected in TRIzol at the ring (10–15 hpi), trophozoite (25–30 hpi), and schizont (40–45 hpi) stages during the next cycle, respectively. Total RNA was extracted with the kit (Zymo Research, USA) according to a standardized procedure. Library preparation for strand-specific RNA-seq was carried out by poly(A) selection with the KAPA mRNA Capture Beads (KAPA) and fragmentation to about 300–400 nucleotides (nt) in length according to the KAPA Stranded mRNA-Seq Kit (KK8421). Libraries were sequenced on an Illumina NovaSeq 6000 system to generate 150 bp pair-end reads.

RNA-seq reads were trimmed by trim-galore, then aligned to the PlasmoDB-45_Pfalciparum3D7_Genome using Hisat2. Read counts were obtained using FeatureCounts. Subsequently, DESeq2 quantified differentially expressed genes (DEGs) with the criteria of ≥2-fold alteration and *p*-adj < 0.05. The Gene Ontology (GO) enrichment analysis was performed on PlasmoDB (https://plasmodb.org/plasmo/).

### RIP-seq and data analysis

RIP assays were performed as previously described (Fan et al., 2020). Briefly, about 5 × 10^9^ synchronized ring-stage parasites were collected and lysed by saponin. The resulting parasite pellet was lysed under nondenaturing conditions (50 mM Tris-Cl at pH 7.4, 150 mM NaCl, 1 mM EDTA, 1 mM EGTA, 1% Triton X-100, 1% NP-40, 1× proteinase inhibitors, 0.2 U/μl RNase inhibitor) for 2 h at 4°C with rotation. The supernatant was collected and incubated with 10 µg mouse anti-ty1 antibodies for 3 h, then incubated overnight with protein G magnetic beads at 4°C. The complex was washed twice with IPP500 (500 mM NaCl, 10 mM Tris-Cl at pH 8.0, 0.05% NP-40, 1 × proteinase inhibitor, 0.2 U/µl RNase inhibitor) and once with PBS, then RNA was eluted by TRIzol reagent and extracted by the phenol-chloroform method. The RNA was directly used to prepare strand-specific RNA-seq libraries without poly(A) enrichment. Libraries were sequenced on an Illumina NovaSeq 6000 system using 150 bp pair-end reads.

For RIP-seq data analysis, reads were removed with trim-galore and aligned to the genome by Hisat2. Samtools was used to sort reads. Peaks (*q*-value cut-off < 0.05) were identified using the macs2 call peak command, by comparing the control and the group with the ty1 antibody, using default settings. Enrichment heatmaps and profile plots were generated using the deepTools computeMatrix and plotHeatmap tools. Peak annotation was carried out using ChIPseeker in RStudio.

## Results

### Generation of *pfnsun1* transgenic parasite line

**Figure 1:**
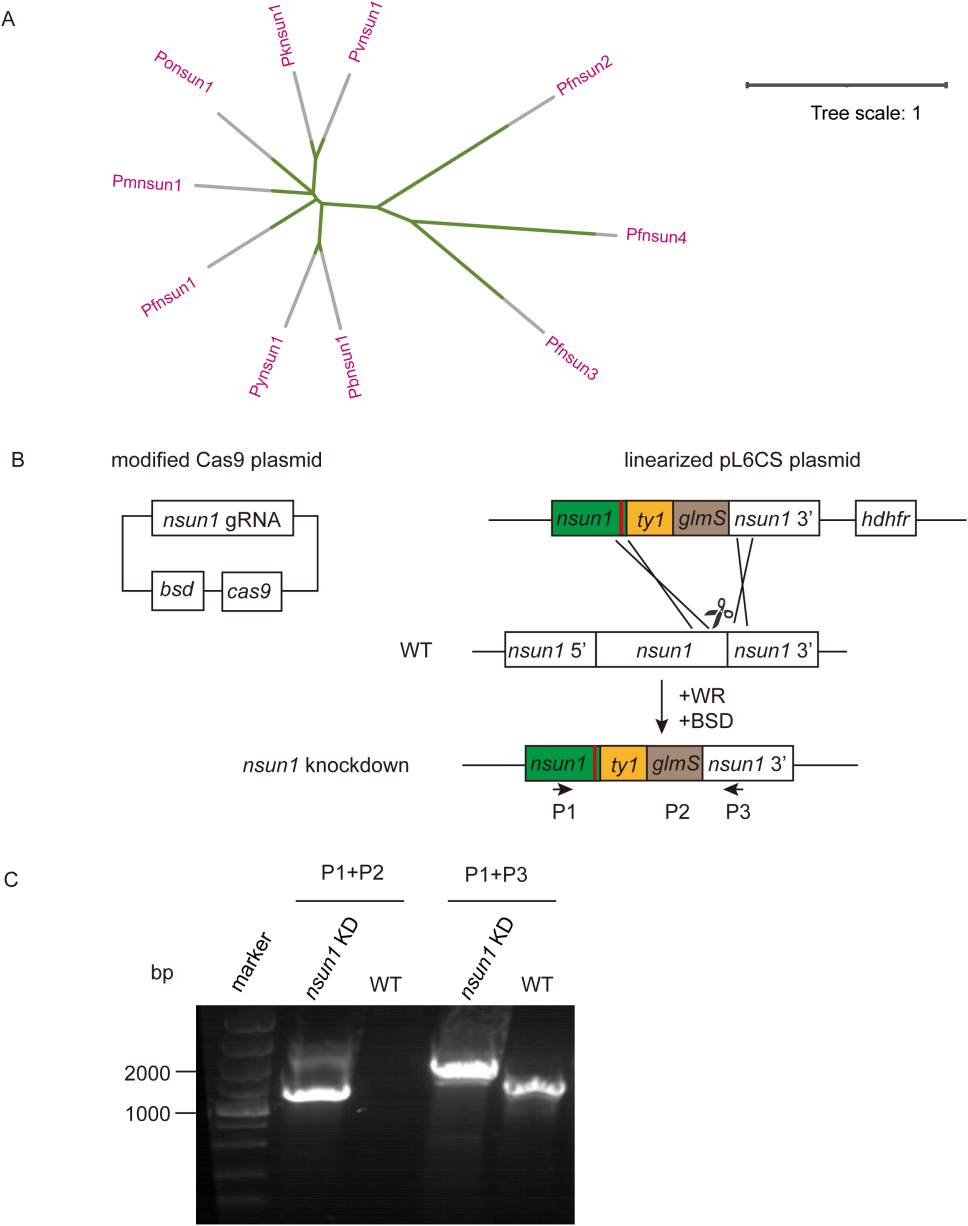
Generation and characterization of *pfnsun1-ty1-glmS* knockdown strain. **(A)** Phylogenetic trees of NSUN protein family orthologs in *Plasmodium* spp. **(B)** Schematic representation of transgenic line *pfnsun1-ty1-glmS* construction. Cotransfection of plasmids pUF1-BSD-cas9 and pL6CS-hDHFR-*pfnsun1-ty1-glmS* leads to gene integration. **(C)** PCR validation of the *pfnsun1-ty1-glmS* line.

*Pfnsun1* (PF3D7_0704200) ORF contains 4,263 bases, encoding a protein of approximately 141.2 kDa in molecular weight and containing one NOP2 structural domain. In order to compare the homology within the NSUN family, we analyzed the amino acid sequences of PfNSUN1 to PfNSUN4, along with other common *Plasmodium* NSUN1 proteins. Our findings showed that sequences of PfNSUN1 had low similarity to PfNSUN2-4 but was conserved among different *Plasmodium* species (**Figure 1A**). To determine the function of *pfnsun1*, we attempted to disrupt the *pfnsun1* gene. However, following three unsuccessful transfection attempts, it was concluded that the gene might be indispensable. Consequently, we opted to construct a knockdown strain. About 1 kb bases before and after the stop codon of the *pfnsun1* gene were selected as homologous arm sequences, and three tandem ty1 tags and a *glmS* sequence were cloned into the plasmid. The constructed plasmids were electro-transferred into wild-type parasites, which were then cultured for about 3 weeks with WR and BSD drug selection until live parasites were observed by microscopy (**Figure 1B**). To confirm successful transfection, we designed a forward primer P1, upstream of the 5′ homologous arm, a reverse primer P2 within the *glmS* sequence, and another reverse primer P3 within the 3′ homologous arm sequence, respectively. The sequence lengths of wild-type and transgenic strains were verified using the P1+P2 and P1+P3 primers. Agarose gel electrophoresis results confirmed the successful generation of the *pfnsun1-ty1-glmS* parasite strain (**Figure 1C**).

### *Pfnsun1* is required for parasite development in the intraerythrocytic developmental cycle

**Figure 2:**
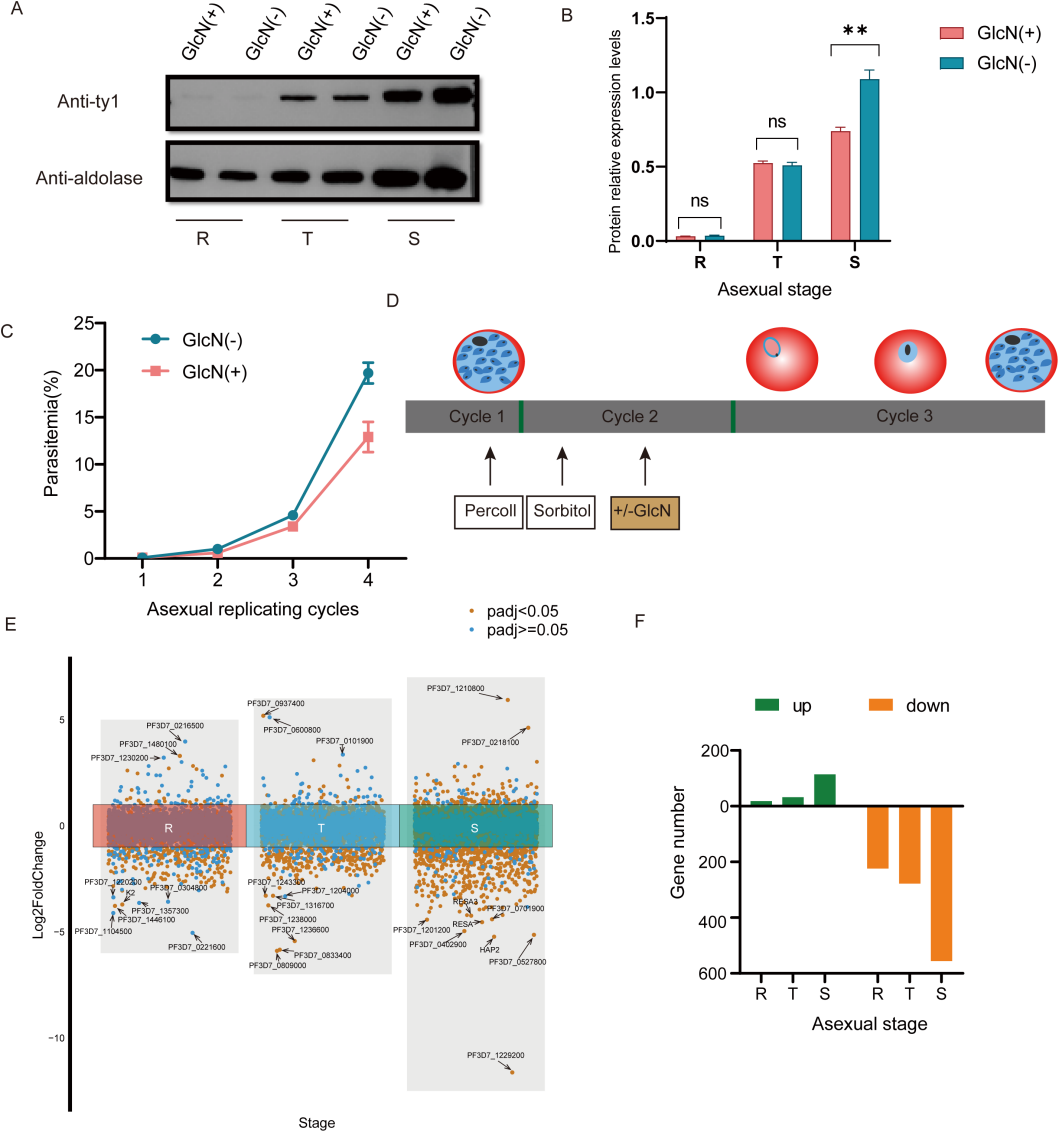
PfNSUN1 protein is required during intraerythrocytic development. **(A)** Western blot of total protein extracts from ring (R), trophozoite (T), and schizont (S) stages from the *pfnsun1-ty1-glmS* strain with or without GlcN. **(B)** Gray value analysis of Western blot experiment. **P< 0.01, ‘ns’: not significant (unpaired two-tailed Student’s t-test). **(C)** Growth curve assay of the *pfnsun1-ty1-glmS* strain with or without GlcN in the culture (*n* = 3, bars are SD). **(D)** Schematic of transcriptome samples collected. The strain experienced Percoll and sorbitol synchronization and was collected at the third cycle with or without GlcN. **(E)** Scatterplot of all genes in the RNA-seq data. Ten genes with the strongest changes are labeled. **(F)** Histogram displaying the number of up or downregulated DEGs.

To verify the knockdown effect of the *pfnsun1-ty1-glmS* transgenic strain, we performed a Western blot assay. The addition of 5 mM GlcN had no significant impact on the growth of wild-type *P. falciparum* parasites as confirmed by previous studies (Liu et al., 2020; Shang et al., 2021b). Thus, we collected the ring, trophozoite and schizont stage samples in the second growth cycle with or without GlcN to extract protein after synchronizing the parasites. The results showed insignificant changes in protein levels at ring and trophozoite stages but a significant decrease at the schizont stage (**Figure 2A**). The gray value of protein electrophoresis with GlcN treatment at the schizont stage decreased by approximately 34% compared to the untreated group (**Figure 2B**). To investigate the importance of *PfNSUN1* protein in the intraerythrocytic developmental cycle, we performed growth curve experiment. We minimized the growth window of the transgenic parasite line to 5 h after rigorous synchronization. When parasites reached the early trophozoite stage, we diluted the parasitemia to 0.1% and continued cultivating for four growth cycles with or without GlcN and counted parasitemia. The results showed that at the fourth cycle, parasitemia of the group without GlcN treatment reached an average of 19.7%, while parasitemia of the group with drug treatment reduced to 12.9%, indicating an approximate 35% decrease in growth efficiency (**Figure 2C**). These results suggest that the *pfnsun1* gene plays an important role in the intraerythrocytic developmental cycle.

### *Pfnsun1* knockdown altered the global transcriptome

To explore the function of PfNSUN1 protein as an epigenetic regulator, we performed strict synchronization and divided the samples into GlcN-added and non-GlcN-added groups. Transcriptome samples were collected in the subsequent growth cycle (**Figure 2D**). RNA-seq data revealed significant transcriptomic changes following *pfnsun1* knockdown. Specifically, there were only 18, 32, and 114 upregulated genes, but 224, 278, and 556 downregulated DEGs at the ring, trophozoite, and schizont stages, respectively (**Supplementary Table S1**). We highlighted 10 genes with the greatest changes at each stage and found that most of the genes were antigenic variant genes or genes with unknown functions. Notably, *K2* was significantly downregulated at the ring stage, and *hap2*, *resa*, and *resa3* were significantly downregulated at the schizont stage. In light of these findings, PfNSUN1 may be regarded as a positive regulator that facilitates gene expression (**Figures 2E, F**).

**Figure 3:**
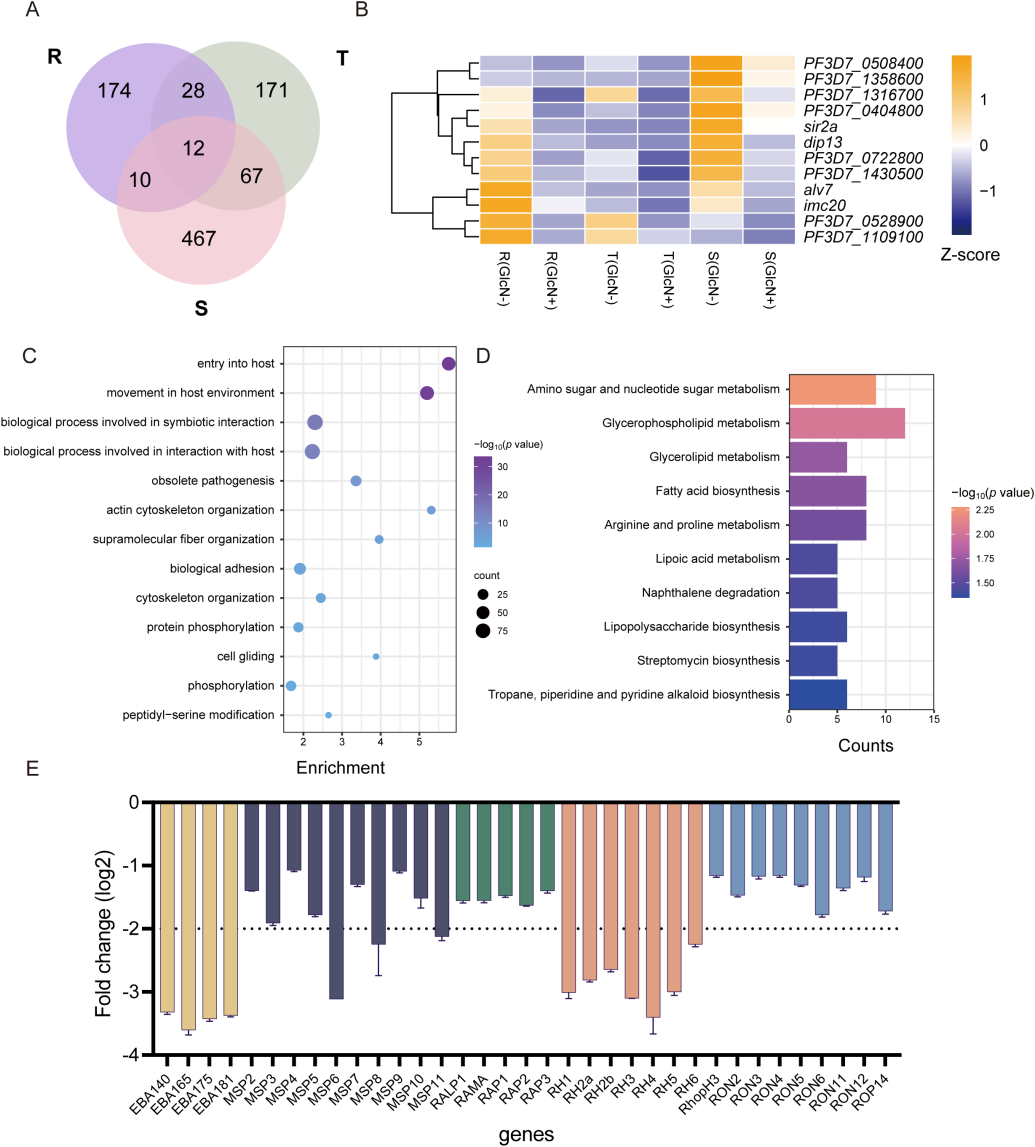
Functional analysis of downregulated DEGs in gene expression regulation. **(A)** Venn diagram showing genes downregulated at R, T, and S stages. **(B)** Heatmap showing the 12 downregulated gene expressing levels of the *pfnsun1-ty1-glmS* strain with or without GlcN across the R, T, and S stages. **(C)** Enriched Gene Ontology (biological processes) terms for the DEGs in the *pfnsun1* knockdown line at the schizont stage. **(D)** Enriched KEGG terms for the DEGs in the *pfnsun1* knockdown line at the schizont stage. **(E)** The log2 fold change (GlcN(+)/GlcN(-)) of down-regulated invasion genes at schizont stage.

To clarify the genes affected by *pfnsun1* knockdown, we took the intersection of down-regulated differentially expressed genes (DEGs) at ring, trophozoite and schizont stages. This analysis revealed 12 genes with consistently decreased expression across all stages (**Figure 3A**). These 12 genes included *sir2a*, *dip13*, *alv7*, and *imc20*. *Sir2a* is an important regulator in *P. falciparum* and is critical for maintaining a stable heterochromatin environment and exclusive expression of antigenic variant genes (Petter et al., 2011; Mancio-Silva et al., 2013) (**Figure 3B**). Given that the schizont stage exhibited the highest number of downregulated DEGs, we selected these genes for GO enrichment analysis. The analysis indicated that these genes impact multiple pathways, including host entry, movement within the host environment, biological process involved in symbiotic interaction, obsolete pathogenesis, actin cytoskeleton organization, protein phosphorylation, peptidyl−serine modification, etc. (**Figure 3C**). To ensure comprehensive pathway identification by GO analysis, we also performed KEGG enrichment analysis on these genes and found that these genes were involved in the regulation of amino sugar and nucleotide sugar metabolism, glycerophospholipid metabolism, glycerolipid metabolism, fatty acid biosynthesis, and other pathways (**Figure 3D**). To ascertain the impact of *PfNSUN1* on parasite growth, we investigated the downregulation of *eba, msp, rap, rh*, and *ron* invasion genes. Our findings revealed that the transcripts of *eba* and *rh* family members exhibited a downregulation of over 4-fold (**Figure 3E**). It was plausible that *PfNSUN1* influenced the expression of these invasion-related genes. In conclusion, these findings indicate that the reduction in parasitemia may be associated with conserved biological processes, such as invasion and metabolism.

### PfNSUN1 binds on 28S ribosomal RNA

**Figure 4:**
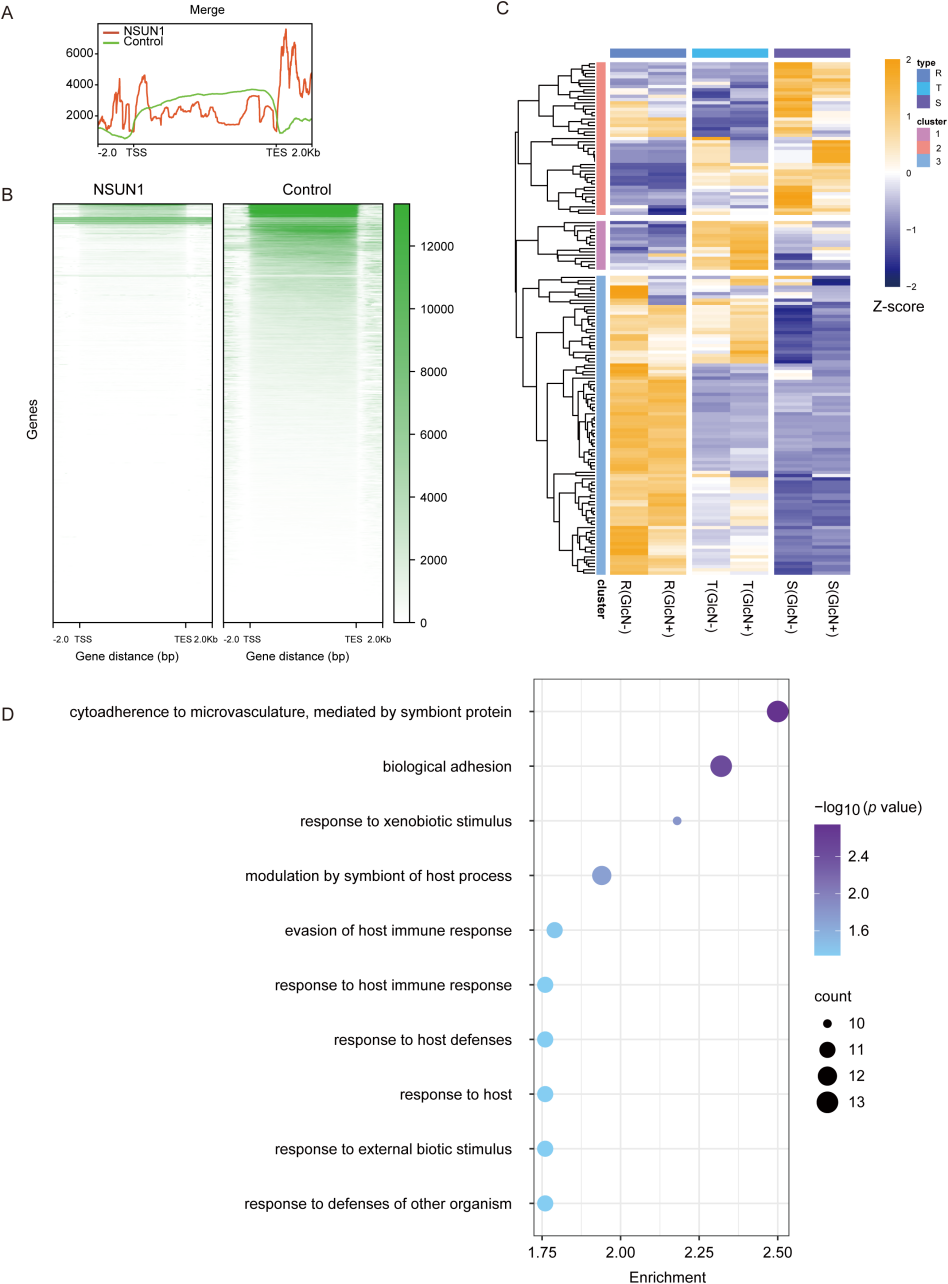
Target gene analysis of PfNSUN1 protein-enriched. **(A)** The average profile of RIP/control enrichment at gene coding sequence. **(B)** Heatmap showing RIP/control fold enrichment at gene coding sequence. TSS, translation start site; TES, translation end site. **(C)** Expressing heatmap of target genes obtained from RIP-seq at ring stage. **(D)** Enriched Gene Ontology (biological processes) terms for the target genes obtained from RIP-seq.

*Pfnsun1* is an RNA methylation factor that influences gene expression by modifying RNA. Therefore, we collected a ring stage sample of the *pfnsun1-ty1-glmS* strain to identify its direct-binding RNAs by RIP-seq experiments. The results showed that RNAs bound by the PfNSUN1 protein were distributed across all 14 chromosomes, as illustrated in the peak diagram (**Figures 4A, B**). There were 154 target genes confirmed after aligning to the genome (**Supplementary Table S2**). In combination with RNA-seq data, our findings indicated that these genes were predominantly down-regulated, thereby substantiating the hypothesis that *PfNSUN1* exerted a positive regulatory effect on gene expression (**Figure 4C**). GO enrichment analysis showed that these genes were involved in pathways including cytoadherence to the microvasculature, biological adhesion, response to xenobiotic stimulus, modulation by symbiont of host process, evasion of host immune response, response to host immune response, response to host defenses, response to host, response to external biotic stimulus, and response to defenses of other organisms (**Figure 4D**). These results showed that PfNSUN1 is related in the response between parasite and host.

**Figure 5:**
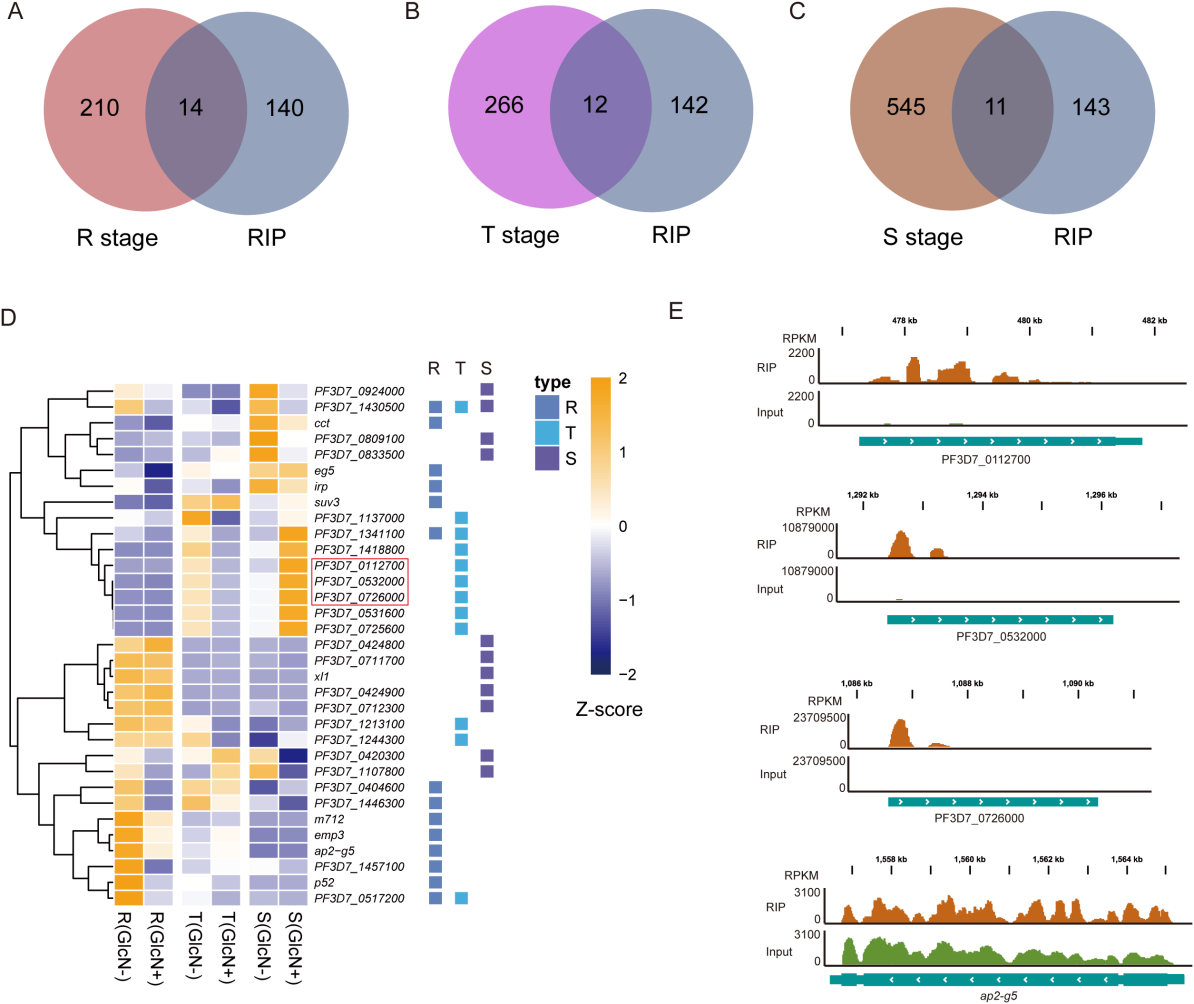
The PfNSUN1 protein modifies the 28S rRNA and *pfap2-g5* transcription factor. **(A–C)** Venn diagrams showing the intersections between target genes from RIP-seq data and downregulated genes at the R, T, and S stages. **(D)** Heatmap showing downregulated target gene expressing levels of *pfnsun1-ty1-glmS* strain with or without GlcN at ring, trophozoite, and schizont stages. 28S rRNAs are marked with a red box. **(E)** Track view showing the enrichment signals of the PfNSUN1 protein on 28S rRNA and *pfap2-g5* transcription factor.

To validate target genes on which PfNSUN1 protein has a significant effect, we took intersections of target genes obtained from RIP-seq data with down-regulated DEGs at different stages. The down-regulated DEGs at ring, trophozoite, and schizont stages had 14, 12, and 11 same genes with PfNSUN1 protein target genes, respectively (**Figures 5A–C**). At ring stage, *pfnsun1* mainly regulates *cct*, *eg5*, *irp*, *suv3*, *m712*, *emp3*, *ap2-g5*, *p52*, etc. The functions of *suv3* and *m712* are unknown. PfCCT has a key regulatory function in the second and rate-limiting step of the *de novo* phosphatidylcholine biosynthesis, which is essential for parasite survival (Izrael et al., 2020). PfEG5 is a molecular motor that cross-links microtubules, similar to other organisms (Cook et al., 2021). PfIRP regulates parasite environment homeostasis by binding iron response elements (Hodges et al., 2005). *Pfemp3* encodes erythrocyte membrane protein 3, which appears on the cytoplasmic surface of the host cell membrane in the later stages and is associated with membrane skeleton and cytoadherence (Waterkeyn et al., 2000). An important factor affected by *pfnsun1* is *pfap2-g5*, which belongs to the largest ApiAP2 transcription factor family in *P. falciparum*. A previous study revealed that *pfap2-g5* is a transcriptional repressor that co-regulates gametocyte development with *pfap2-g* (Kafsack et al., 2014; Poran et al., 2017; Shang et al., 2021a) (**Figures 5D, E**). Additionally, we found that other ApiAP2 transcription factors showed a decrease in transcript level after *pfnsun1* knockdown, such as *ap2-o*, *PF3D7_0420300*, *PF3D7_1107800*, etc. (**Supplementary Figure S1**). PfP52 is a member of the 6-cysteine family, which can ensure that sporozoites enter hepatocytes (Arredondo and Kappe, 2017). Target genes such as *xl1* were downregulated at the schizont stage (**Figure 5D**). PfXL1 is possibly associated with adhesion between parasites and erythrocytes (Spillman et al., 2016). Noteworthy, *PfNSUN1* knockdown caused some ncRNA down-regulated at trophozoite stage, most of them were 28S rRNA, such as *PF3D7_0112700*, *PF3D7_0532000*, and *PF3D7_0726000* (**Figures 5D, E**). The results indicated that PfNSUN1 is involved in ribosome biogenesis, which is consistent with human NSUN1 protein (Liao et al., 2022).

## Discussion

The NSUN family is an important m^5^C-modified factor in eukaryotes and plays an important role in gene expression regulation. Recent studies have increasingly focused on this family, particularly concerning human-related tumorigenesis and progression, such as hepatocellular carcinoma and bladder cancer (Awah et al., 2021; Wang et al., 2023; Zhang et al., 2023; Gu et al., 2024). A recent study demonstrated that NOP2/NSUN1 catalyzes the deposition of m^5^C at position 4447 on 28S rRNA and regulates ribosome biogenesis through noncatalytic complex formation with box C/D snoRNPs (Liao et al., 2022). In yeast, the NSUN protein homolog, NOP2, has the ability of m^5^C modification on 25S rRNA (Sharma et al., 2013). In *Plasmodium* spp., four genes (NSUN1–4) are predicted to contain the NOP2 functional domain. Currently, the RNA modification map of NSUN2 is the only one that has been characterized. Disrupting the gene encoding NSUN2 in *P. yoelii* results in a complete loss of gametocyte generation, while *P. falciparum* shows very low gametocyte productivity (Liu et al., 2022). Thus, the NSUN family is indispensable for parasite growth and transmission, contributing to gene regulatory diversity.

In this study, we focus on exploring the role of *pfnsun1* in gene expression regulation. Despite several unsuccessful knockout attempts, we constructed an inducible knockdown system. The knockdown efficacy of this strain reached only about 34%, which was not a perfect knockdown strain, but we already could observe a genome-wide effect brought by *PfNSUN1*. The majority of DEGs exhibited down-regulation, while a subset demonstrated up-regulation. Since *pfnsun1* knockdown altered the expression level of regulatory factors such as *sir2a*, *pfap2-g5*, *pfap2-o*, etc., it is unclear whether *pfnsun1* regulates these genes directly or indirectly. In addition, we obtained 154 target genes directly bound by the PfNSUN1 protein. In other eukaryotes, the NSUN1 protein was identified to modify 28S rRNA and 25S rRNA (Sharma et al., 2013; Liao et al., 2022). In this study, PfNSUN1 protein was significantly enriched at several genes encoding 28S rRNA, suggesting it is conserved in regulating ribosome biogenesis. Furthermore, we found that PfNSUN1 protein was highly enriched at the activated *var* gene, indicating a potential association with parasite virulence (Scherf et al., 2008; Maier et al., 2009). Transcriptional data show a high transcript abundance of *pfnsun1* at the ring stage, trophozoite stage, gametocyte V and ookinete stage (López-Barragán et al., 2011) (**Supplementary Figure S2**). Thus, PfNSUN1 protein may be involved in other critical life processes, such as gametocyte production, by regulating m^5^C modification of *pfap2-g5*, though this speculation requires further verification.

In summary, our data reveal that PfNSUN1 protein is required during IDC in *P. falciparum*, playing a significant role in regulating ribosome biogenesis and gene expression. These findings provide new insights into malaria control and potential drug discovery.

## Data Availability

The datasets presented in this study can be found in online repositories. The high-throughput sequencing data of this study have been deposited in Gene Expression Omnibus (GEO) database under accession number GSE254642 and GSE254643/**Supplementary Material**.

## Supplementary material

Supplementary Figure 1 Heatmap of transcription levels of ApiAP2 transcription factor family with or without GlcN.

Supplementary Figure 2 Dynamic changes of *pfnsun1* transcript abundance (TPM) during the whole life history (López-Barragán et al., 2011).
