# Supplementary figures and images for: The RNA m5C methyltransferase NSUN1 modulates human malaria gene expression during intraerythrocytic development
Source: Front Cell Infect Microbiol. 2024 Oct 7;14:1474229. doi: 10.3389/fcimb.2024.1474229 (PMC11491294; doi:10.3389/fcimb.2024.1474229)

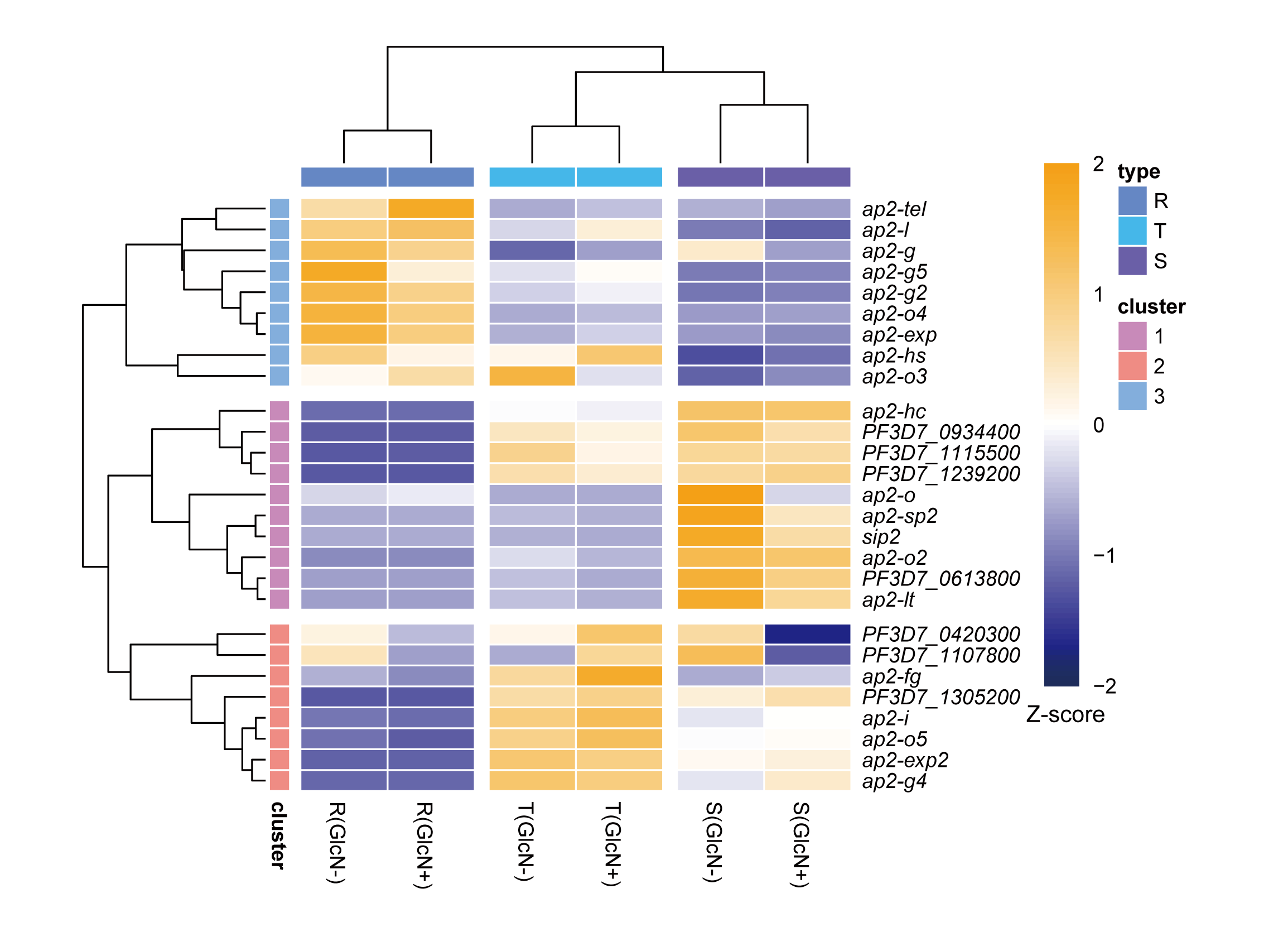

Supplement: Supplementary Figure 1 — Heatmap of transcription levels of ApiAP2 transcription factor family with or without GlcN. [file Image1.tif]

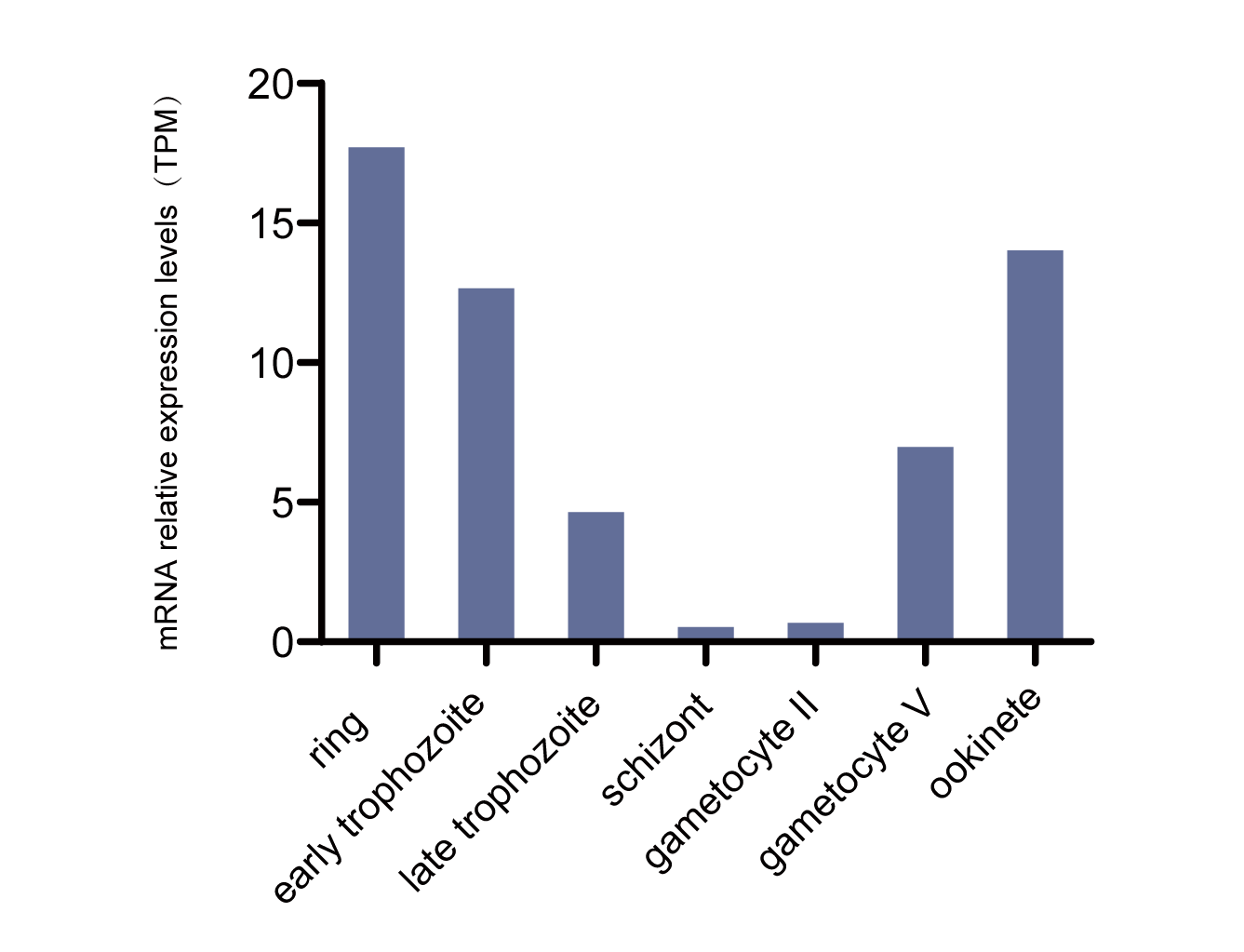

Supplement: Supplementary Figure 2 — Dynamic changes of pfnsun1 transcript abundance (TPM) during the whole life history (López-Barragán et al., 2011). [file Image2.tif]
